# An unusual case of femoral arteriovenous fistula associated with acute limb ischemia following femoral vein catheterization for hemodialysis

**DOI:** 10.1590/1677-5449.202101992

**Published:** 2022-05-02

**Authors:** Thushan Gooneratne, Kankam Arrachchige Nuwan Chanaka, Serosha Mandika Wijeyaratne

**Affiliations:** 1 University of Colombo, Colombo, Sri Lanka.; 2 Post Graduate Institute of Medicine, Colombo, Sri Lanka.

**Keywords:** vascular access devices, dialysis, femoral artery, ischemia, arteriovenous fistula, vascular surgical procedures, dispositivos de acesso vascular, diálise, artéria femoral, isquemia, fístula arteriovenosa, procedimentos cirúrgicos vasculares

## Abstract

Co-occurrence of acute limb ischemia (ALI) and arteriovenous fistula (AVF) as a manifestation of inadvertent arterial injury during percutaneous femoral vein dialysis catheter insertion is a rare and dangerous, but preventable complication. Iatrogenic femoral AVF commonly presents late, with leg swelling or high output cardiac failure. However, the co-occurrence of a femoral AVF with both progressive leg swelling, and acute thromboembolism has not been previously reported. We report the case of an iatrogenic femoral AVF with superficial femoral artery (SFA) thrombosis and distal embolism in a 53-year-old female who underwent percutaneous femoral access for temporary hemodialysis. Both the SFA and AVF were managed with open surgical repair.

## Resumo

A coocorrência de isquemia aguda de membro (IAM) e fístula arteriovenosa (FAV) como uma manifestação de lesão arterial inadvertida durante a inserção de cateter para hemodiálise por via femoral percutânea é uma complicação rara e perigosa, porém evitável. A FAV femoral iatrogênica geralmente tem apresentação tardia, com edema dos membros inferiores ou insuficiência cardíaca de alto débito. No entanto, a coocorrência de FAV femoral com edema progressivo dos membros inferiores e tromboembolismo agudo não foi previamente relatada. Relatamos o caso de uma FAV femoral iatrogênica com trombose da artéria femoral superficial (AFS) e embolia distal em uma paciente do sexo feminino, 53 anos, submetida à acesso femoral percutâneo para hemodiálise temporária. Tanto a AFS quanto a FAV foram manejadas com reparo cirúrgico aberto.

## INTRODUCTION

Iatrogenic femoral arteriovenous fistula (AVF) is a rare complication of percutaneous femoral access for temporary extracorporeal renal replacement therapy.^1^ The incidence of post-catheterization AVF ranges from 0.006 to 0.86%.^2^ The majority present late with chronic limb swelling, high output cardiac failure, and rarely deep vein thrombosis,^3^ whilst approximately 30% of such post cannulation iatrogenic femoral AVFs resolve spontaneously. When there is a concomitant injury to the arterial intima, dissection may occur causing partial or complete arterial occlusion with resultant acute limb ischemia.^4^

Presentation of an iatrogenic femoral AVF with concomitant limb swelling and distal thromboembolism has not been previously reported. Management of such patients is complex and involves percutaneous endovascular methods or open surgery.

We report a case of an iatrogenic femoral AVF following placement of a dialysis femoral line that presented acutely with limb swelling, distal thromboembolism, and groin sepsis after its removal. We highlight the mechanism of this injury, the need for vigilance for such complications, and how it might be prevented.

The study was conducted in accordance with the relevant standards of the institutional ethics committee and the Helsinki declaration. Informed, written consent was obtained from the patient prior to data collection and publication of the case report.

## CASE DESCRIPTION

A 53-year-old female diagnosed with end-stage renal failure secondary to diabetes mellitus and hypertension was awaiting maturation of her recently created brachiocephalic AVF. She presented to the district hospital with the clinical picture of fluid overload, necessitating urgent dialysis. A double lumen dialysis catheter was inserted into the left femoral vein. The neck veins were avoided due to the presence of the brachiocephalic AVF. She underwent dialysis via the left femoral catheter for two weeks but subsequently developed a purulent peri-catheter discharge and bleeding that necessitated its removal.

**Figure 1:**
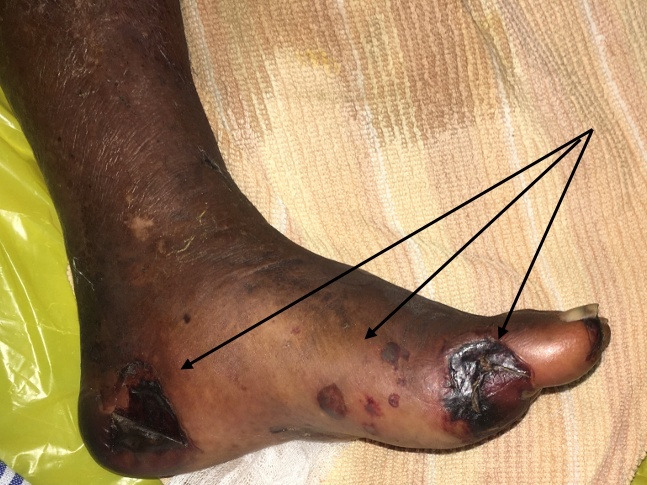
Discrete patchy gangrenous lesions in the left foot in keeping with distal thromboembolism ‘Trash foot’ (black arrows).

Removal of the dialysis catheter was followed by rapid swelling of the entire lower extremity and a diagnosis of acute ileo-femoral deep vein thrombosis was suggested. Approximately 24-36 hours afterwards, she developed acute limb ischemia with multiple gangrenous patches in her foot and toes suggestive of a ‘trash foot’ (Figure 1), prompting a vascular referral.

She was hemodynamically stable, but the entire lower extremity was severely swollen, cold, numb, and paralyzed (her left knee and ankle joints were graded 1 on the Medical Research Council muscle power scale^5^). She had a palpable femoral pulse, but the popliteal and pedal pulses were absent.

**Figure 2:**
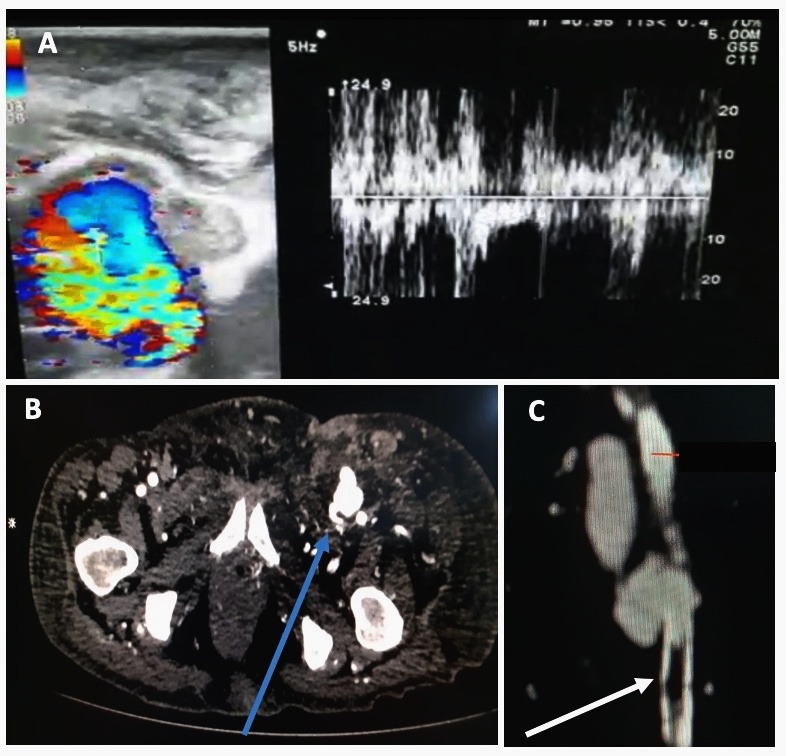
(A) Duplex ultrasonography demonstrating the characteristic turbulent spectral pattern of an arteriovenous fistula (AVF); (B) Computed tomography angiography (axial) demonstrating the presence of high-density contrast in femoral vein suggestive of an AVF (blue arrow); (C) Computed tomography reconstructed image of left superficial femoral artery (SFA) indicating the presence of thrombus in the SFA (white arrow).

Both Duplex ultrasound and CT angiography confirmed an AVF between the right superficial femoral artery (SFA) and femoral vein. Imaging further revealed a thrombus in the SFA just distal to the AVF (Figure 2). However, the distal vascular tree remained patent down to the foot on CT.

**Figure 3:**
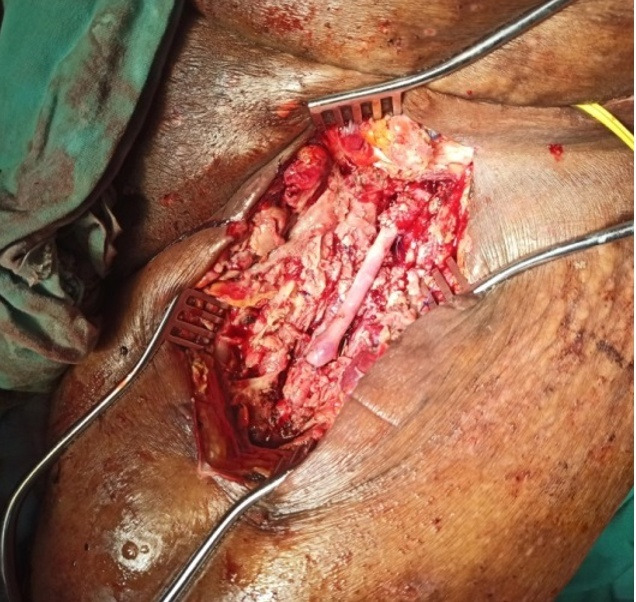
Operative images of the left superficial femoral artery reconstructed with an interposition vein graft after discontinuing the arteriovenous fistula.

Owing to the presence of intraluminal thrombus in the SFA, open surgical thrombectomy and vessel repair was embarked upon after confirming the viability of muscles at fasciotomy. Following femoral vascular control, the AVF was discontinued, and the femoral vein was closed primarily. The catheter-inflicted SFA injury was such that a 4 cm segment was excised and repaired with an interposed reversed vein graft (Figure 3). Postoperatively, she became severely septic, the groin wound dehisced, and the vascular repair was disrupted necessitating common femoral ligation and high above knee amputation. Comorbidities and uncontrolled systemic sepsis resulted in her death 3 weeks later.

## DISCUSSION

Although the incidence of femoral AVF following cardiac catheterization has been reported at 0.86 – 1.3%,^2^ its incidence following femoral hemodialysis catheter insertion is not known. Maruyama et al.^6^ described a case of AVF involving the femoral vessels after a dual lumen hemodialysis catheter in 2002 and there have been a few similar case reports since.^7^^,^^8^

Among many factors known to increase the risk of femoral access AVF,^9^ most were recognized in our patient. These include female gender, hypertension, ‘blind’ landmark guided puncture, left groin puncture by a right-handed operator, and use of heparin.

The narrow vessel diameter among females is likely to predispose to more difficult access and combined with the driving force of elevated blood pressure may promote AVF formation. The left groin puncture by a right-handed clinician alters the accustomed angle of needle trajectory predisposing to simultaneous puncture of artery and vein. A large diameter catheter and anticoagulation therapy with heparin would also have contributed. It was unclear whether ultrasound guidance was employed during insertion of the catheter, however, a too low puncture of the SFA, as in our case, is a known predictor of AVF formation. The use of real-time ultrasound significantly reduces the risk of access-related complications when compared to the traditional landmark insertion technique.

The presentation of femoral AVFs is varied. They often remain unnoticed for a long time and present late with chronic limb swelling, features of steal syndrome, aneurysmal degeneration of the artery, and high output cardiac failure.^3^ An acute presentation with progressive limb swelling, limb pain, and thromboembolism as in our case is extremely rare. The combination of venous hypertension and lymphatic damage would have contributed to the leg swelling. The pattern of neurological deficit and the patency of macro-vessels pointed to distal thromboembolism rather than acute arterial occlusion. The inadvertent SFA intimal injury would have resulted in a non-occlusive local thrombus that subsequently embolized downstream.

Owing to its rarity, there are no clear guidelines on the management of femoral AVF, and thus its management should be individualized. Diagnosis is based on clinical suspicion and confirmed by imaging. Watchful waiting and minimally invasive methods have been advocated as first-line therapeutic options.^10^^,^^11^ Spontaneous closure has been reported in up to 30% of femoral AVF^3^ but is less likely in patients on anti-platelets and anti-coagulation therapy.^10^ In our case, acute limb ischemia demanded an immediate intervention to clear the distal thrombus and repair the vessels.

The use of percutaneous minimal femoral vessel access is gaining in popularity and is frequently employed in cardiovascular diagnostic and therapeutic procedures, as well as for temporary vascular access for patients requiring hemodialysis. Our case highlights the need for awareness of the rare complication of femoral AVF and the addition of acute arterial thromboembolism. The authors highlight the importance of prevention of inadvertent arterial injury by routine use of real-time ultrasound over the traditional landmark guided insertion.

Finally, nephrologists and dialysis staff must be aware of this rare but dangerous complication and the need for timely surgical referral.

## CONCLUSION

Iatrogenic femoral arteriovenous fistula associated with acute arterial thromboembolism is a rare, but potentially avoidable, serious complication following femoral vein catheterization for hemodialysis. Awareness of methods to prevent inadvertent arterial injury and prompt diagnosis and referral are emphasized to minimize morbidity and mortality.
